# Science, society and biosafety of a field trial with transgenic biofuel poplars

**DOI:** 10.1186/1753-6561-5-S7-I23

**Published:** 2011-09-13

**Authors:** Rebecca Van Acker, Véronique Storme, Geert Goeminne, Bart Ivens, René Custers, Dirk Aerts, Wim Soetaert, John Ralph, Nicholas Santoro, Jean-Charles Leplé, Gilles Pilate, Wout Boerjan

**Affiliations:** 1 Department of Plant Systems Biology, VIB, Gent, Belgium; 2 Department of Plant Biotechnology and Genetics, UGent, Gent, Belgium; 3 VIB Headquarters, Gent, Belgium; 4 Department of Biochemical and Microbriol Technology, UGent, Gent, Belgium; 5 D.O.E. Great Lakes Bioenergy Research Center, U. of Wisconsin, Madison, WI, USA; 6 D.O.E. Great Lakes Bioenergy Research Center, Michigan State U., East Lancing, Michigan, USA; 7 INRA Centre d'Orléans, Unité Amélioration, Génétique, et Physiologie Forestières, Orleans cedex 2, France

## Background

Global warming, environmental disasters, and increasing oil prices have catalyzed a worldwide trend to use plant biomass as a renewable source for liquid biofuels and bio-based materials. Plant biomass can be processed into fermentable sugars by enzymatic depolymerization of the cell wall polysaccharides, followed by fermentation. However, the presence of lignin in the cell wall constitutes a major recalcitrance factor because it limits the accessibility of polysaccharidases to the cellulose microfibrils. To overcome this hurdle, plant biomass is pretreated in a costly and energy-requiring process. One approach to overcome the recalcitrance problem is to engineer lignin amount or alter its composition to make lignin more susceptible to chemical degradation [1]. Cinnamoyl-CoA reductase (CCR), the enzyme that converts feruloyl-CoA into coniferaldehyde, is considered the first enzyme in the monolignol-specific branch of the phenylpropanoid pathway. Poplar trees down-regulated in CCR have been produced in the early nineties and planted in a field trial in France to produce sufficient wood for small scale chemical pulping tests [2]. These trees had 20% lower lignin levels and relatively more cellulose per gram of wood [2]. Given that lignin is one of the main limiting factors limiting the conversion of plant biomass into fermentable sugars, and that poplar is considered as a promising second generation biofuel crop, we have re-grown these trees in the greenhouse and in the field, and evaluated wood produced from these trees by saccharification experiments.

## Methods

Cinnamoyl-CoA reductase (CCR) expression was down-regulated in poplar by sense and antisense strategies [2]. Transgenic trees were evaluated for lignin amount and composition [2] and for sugar release by saccharification assays [3]. After obtaining permission from the regulatory authorities, two transgenic lines were planted in a field trial in Belgium and two in a field trial in France, both under short rotation coppice culture to maximize biomass production. Wood was saccharified with and without acid pretreatment.

## Results

Wood from CCR down-regulated trees that were grown in the greenhouse was saccharified with and without acid pretreatment. Interestingly, wood from the transgenic lines released more glucose in saccharification assays performed without pretreatment than wood from wild type trees when saccharified with a pretreatment, demonstrating the improved processing of the cell wall polysaccharides by the enzyme cocktail. These promising results prompted us to ask permission for a field trial to verify whether wood from these trees, when grown in an open field, would still be more easily saccharified. After all, when trees are grown in the greenhouse, they do not experience wind, rain and seasons, and they need to be stacked to grow upward. After a one year long calvary to obtain regulatory permission for the Belgian field trial [4], the trees were grown under short rotation coppice culture in replicated clonal blocks in a small plot of a total of ~ 500 trees. The plot has a fence and is surveilled by cameras. In Spring, the trees are regularly monitored for flowering as they are not allowed to flower. Flowering is not expected because the coppice cycles are as short as 3 years. The possible effects of the low lignin trait on rust infection and insects is being monitored, but it is not expected that these effects will be larger than those associated with conventionally bred poplar hybrids or will be specific to the transgenic trees taking the large genetic diversity of the wild poplar germplasm into account. Activist groups that are concerned that these trees pose different risks than conventionally bred hybrids or pose a risk for human health are invited for a discussion with scientists and the biosafety manager at VIB.

After one year of growth in the field, trees were coppiced to allow the development of multiple shoots in the next spring. The basal 20 cm part of the stem was debarked. Wood harvested from wild type trees was white in appearance, but wood from the transgenic trees was red-brown, most likely due to reactions with ferulic acid, derived from the substrate of CCR, in the cell wall [5]. Importantly, the red coloration was not uniform, but varied significantly in a single trunk, and among trunks from clonally propagated trees. Saccharification potential increased to ~50% per gram dry weight.

Our data show that reducing lignin amount in trees improves saccharification potential and should reduce the chemical or energy cost of biomass pretreatments. The trees will be harvested again in winter 2012-2013, i.e. three years after the first coppice. This should provide sufficient biomass for evaluation at semi -industrial scale at the pilot biorefinery in the Port of Ghent[6].
